# Discrimination of human papillomavirus genotypes using innovative technique nested-high resolution melting

**DOI:** 10.1038/s41598-022-14730-9

**Published:** 2022-08-17

**Authors:** Melika Alirezaei, Sayed Hussain Mosawi, Ali Afgar, Mehdi Zarean, Tahereh Komeili Movahhed, Vajiheh Abbasi, Reza Fotouhi-Ardakani

**Affiliations:** 1 Cellular and Molecular Research Center, Qom University of Medical Sciences, Qom, 3736175513 Iran; 2 Medical Sciences Research Center, Ghalib University, Kabul, Afghanistan; 3 Research Center for Hydatid Disease in Iran, Kerman University of Medical Sciences, Kerman, Iran; 4 Department of Parasitology and Mycology, School of Medicine, Mashhad University of Medical Sciences, Mashhad, Iran; 5 Department of Medical Biotechnology, School of Medicine, Qom University of Medical Sciences, Qom, Iran

**Keywords:** Biological techniques, Biotechnology, Genetics, Microbiology, Molecular biology, Molecular medicine

## Abstract

The prompt detection of human papillomavirus and discrimination of its genotypes by combining conventional methods in new molecular laboratories is essential to achieve the global call of eliminating cervical cancer. After predicting the melting temperature of an approximately 221 bp region of the *L*1 gene from different HPV genotypes by bioinformatics software, an innovative technique based on the nested- high resolution melting was designed with three approaches and using conventional PCR, qPCR, and diagnostic standards. HPV-positive samples identified by microarray along with diagnostic standards were evaluated by qPCR-HRM and discordant results were subjected to sequencing and analyzed in silico using reference types. In addition to screening for human papillomavirus, nested-qPCR-HRM is one of the modified HRM techniques which can discriminate some genotypes, including 6, 16, 18, 52, 59, 68 and 89. Despite the differences in diagnostic capabilities among HRM, microarray and sequencing, a number of similarities between HRM, and sequencing were diagnostically identified as the gold standard method. However, the bioinformatics analysis and melting temperature studies of the selected region in different HPV genotypes showed that it could be predicted. With numerous HPV genotypes and significant genetic diversity among them, determining the virus genotype is important. Therefore, our goal in this design was to use the specific molecular techniques with several specific primers to increase sensitivity and specificity for discriminating a wide range of HPV genotypes. This approach led to new findings to evaluate the ability of different approaches and procedures in accordance with bioinformatics.

## Introduction

Human papillomaviruses (HPVs) are responsible for various human pathologies, being the primary agent for the second most prevalent cancer among women, cervical cancer^1, 2^. The cofactor role of HPV and other risk factors was proven in the progression of cervical cancer^3^. The genotyping of human papillomaviruses is challenging due to their high diversity. Determining the genotypes of this virus plays an essential role in breaking the infection transmission cycle, treatment management, vaccine development, which will result to avoid cancer occurrence. As mentioned in the study of Bonde JH et al., human papillomavirus genotyping discriminates the risk of cervical intraepithelial neoplasia grade 3 or worsens (CIN3 +) or with more intensity to a clinically significant degree, irrespective of cytology result^4^. Furthermore, Arroyo Mühr, LS, et al. stated that when cervical cancers were analyzed by both PCR and deep sequencing, HPV was present is > 92% of cervical cancer samples^5^.

The severe danger of HPV for women and its relation with different cancers necessarily require genotyping by a susceptible and rapid method^6^.

The genotyping of HPV in various studies was performed via the nucleotide sequencing, DNA Chip, hybridization, reverse line blot (RLB) assays, generic and type-specific probes, liquid bead microarray assay (LBM) based on Luminex technology, etc.^7^.

DNA sequencing is the gold standard assay for the accurate viral typing^8^; whereas microarray provides genotype-specific information which can detect the presence of mixed HPV infection, but both techniques are expensive, and they are not available everywhere. Compared to two methods mentioned above, the hybridization DNA-RNA method could detect 13 high-risk human papillomaviruses, but without their type specificity^9, 10^. RLB assay has a limit in repeatability and is laborious to set up, in addition to the high expenses^10, 11^.

There are other quick and accurate methods, such as probe-based methods, but they are also expensive and cannot be used as a routine screening method due to lack of affordability by the general public^12^.

Because it is challenging to design multiple compatible primer sets for the genotype-specific PCR, the maximum number of human papillomaviruses detectable in a single assay is relatively limited^13^. Thus, the combination of two techniques, qPCR and high-resolution melting (HRM), can be used for genotyping. Moreover, when microarray and sequencing are compared with qPCR and HRM, microarray and sequencing could only detect 250 copies of the virus out of 1000, while qPCR could detect as few as ten copies of the virus^14^.

An advantage of qPCR is that some of the detection chemistries which are used to allow for a post-PCR high-resolution melting curve analysis provide additional information on the specificity^15^. Especially for targets with high homology, such as HPV, additional details of the melting temperature, such as those obtained in real-time PCR, are valuable to reduce false positivity.

HRM method, which decreases costs, could be a suitable replacement for other genotyping methods and a powerful technique to use dye chemistry, resolution instruments, and data analysis^16, 17^. The high sensitivity and specificity HRM, along with the melting temperature prediction, has high importance for different genotype diagnoses; indeed, this technique can distinguish various temperatures with the disputes of 0.2–1.1 from each other^18, 19^.

One of the crucial issues in HRM diagnostic approaches is accurately identifying the gene region among different types of strains. This method, due to the difference in melting temperature alteration of gene region and common primers, could discriminate species and genotypes^20^.

HRM is one of the most powerful methods to study genetic variation such as SNPs, insertions, and/or deletions in the genome. The fundamental aspect of method is that a minor variation in the nucleic acid sequence leads to a detectable alteration in the melting curve, and differences in the amplified PCR sequences^21, 22^.

The advantage of HRM method is the amplification of short DNA fragments with high resolution and accuracy. However, some limitations, such as the mismatch of intercalated dyes to ambiguous sequences, like dimer primers and nonspecific products, should not be ignored^23^. This can lead to missing fragments that have an identical melting point^20^.

This feature has a vital role in the genotyping and other applications, including finding genetic variation, mutation screening, methylation analysis, etc.^17^. The quick diagnosis of human papillomavirus could prevent the development and progression of cervical cancer. Moreover, the determination of viral load in women with positive HPV requires a highly sensitive method^24^. The determination of viral load in women is of great interest because it is associated with disease prognosis. Analysis of human papillomavirus viral load and genotype improves prediction of invasive cervical cancer^25^.

We used molecular methods, including qPCR and HRM, to screen, genotype and discriminate HPV. Our goal is to design a novel approach for a rapid, sensitive, specific, and cost-effective diagnosis of HPV genotype in the cervical cancer samples.

## Results

### DNA analysis and primer design

Cervical tissue DNA was successfully extracted with genomic DNA concentrations ranging from 50 to 387 ng/µl. Moreover, the mean A260/A280 ratio obtained for all DNA samples was approximately 1.82, which indicates pure DNA (mean standard deviation = 0.05). The beta-actin gene was observed for all samples as an internal extraction control with a band size of 120 bp.

Consensus PCR amplifying 486 bp of the HPV *L*1 region using degenerate primers FRG5 and MY09 could detect 100 HPV-positive samples revealing different genotypes. Another primer pair, FRG5/FRG2, amplified 215–221 bp of HPV *L*1 and corresponding amplicons generated up to seven different HPV genotypes (6, 16, 18, 52, 59, 66, and 89).

### Bioinformatics’ analysis

The primary analysis based on Tm and high-resolution melting curves for predicting the melting temperature of 24 different HPV genotypes selected from GenBank showed that while some genotypes could be isolated from each other using bioinformatics analysis, other genotypes showed slight temperature differences in the 215–224 bp HPV *L*1 region (Table 1).

**Table 1:** In the silico prediction of melting temperature of different human papillomavirus genotypes using CLC Genomics Workbench 12 bioinformatics application (salt = 0.2 M).

Predicted	Genotype	Accession	(FRG5/2 primer)		(GP5 + /FRG2 primer)	
Group			Length (bp)	Tm (°C)	Length (bp)	Tm (°C)
1	HPV 35 (H)	MT218006.1	80.58	218	143	79.02
	HPV 73 (pro-H)	KF436834.1	80.66	224	149	79.47
	HPV56 (H)	KX645780.1	80.92	215	140	78.63
	HPV 31 (H)	LR862018.1	80.96	218	143	79.02
2	HPV 42 (L)	HE820175.1	81.30	215	140	80.09
3	HPV 58 (H)	HM639442.1	81.68	215	140	79.21
	HPV 16 (H)	LC647457.1	81.71	218	143	79.59
	HPV 59 (H)	KC470266.1	81.92	221	146	80.59
	HPV68 (H)	KC470283.1	81.92	221	146	79.67
4	HPV 45 (H)	LR862061.1	82.11	221	146	80.79
5	HPV 52 (H)	MK387726.1	82.45	215	140	79.80
	HPV 6 (L)	MK463909.1	82.45	215	140	80.97
	HPV 11 (L)	KU721798.1	82.45	215	140	80.97
	HPV 53 (pro-H)	KU951266.1	82.45	215	140	80.68
	HPV39 (H)	MK344672.1	82.48	221	146	81.08
	HPV 18 (H)	MH057745.1	82.48	221	146	81.64
	HPV 66 (H)	MH607470.1	82.64	215	140	80.97
6	HPV54 (L)	U37488.1	83.02	215	140	80.68
7	HPV 51 (H)	MH577964.1	83.21	218	143	80.74
	HPV 43 (L)	HE962408.1	83.59	221	146	82.76
	HPV 84 (L)	LR861944.1	83.59	215	140	82.43
	HPV 40 (L)	MK463923.1	83.96	221	146	82.76
	HPV 83 (H)	AF151983.1	83.97	215	140	82.73
	HPV 89 (L)	LR861984.1	83.97	215	140	83.02

*H* High risk; *L* Low risk; *pro-H* probably High risk.

### Evaluation of semi-nested qPCR-HRM assay primers for HPV genotyping

Several HRM primers and an unlabeled probe were designed for different HPV genotypes to perform HRM analysis using 3 approaches (Table 2). All 57 samples were amplified using all three approaches to define the Tm at the melting step and discriminate HPV types based on this temperature (Table 3).

**Table 2:**
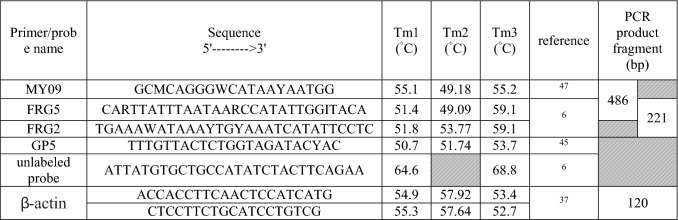
Degenerate primers and unlabeled probes applied for genotyping human papillomaviruses by a high-resolution melting technique.

*Tm1* Melting temperature settings in OligoAnalyser 3.1, qPCR defaultsl; *Tm2* Melting temperature settings in NCBI (Primer Blast); *Tm3* Melting temperature settings by synthesized primer company.

*Unlabeled probes are inexpensive, provide the sequence specificity of probes, and allow simultaneous identification of multiple alleles by melting analysis.

**Table 3:** Comparison of three methods (microarray, sequencing (FRG2/5 primer), and HRM) for genotyping HPV among positive samples.

Row	Sample ID	Result of sequencing			Accession number	Results of microarray	Tm (°C) HRM observed
		Genotype	Query cover (%)	Percent Identity			
1	14 BA	HPV 59	92%	98%	MZ305436	HPV 45	80.5
2	17 BA	HPV 59	100%	100%	MZ305435	HPV 59	80.5
3	20 BA	HPV 59	100%	99.25%	MZ305437	HPV 84	80.4
4	G 11	HPV 59	99%	95.24%	MZ305438	HPV 11	80.3
		HPV 11	100	83.61	MZ305441		
5	G 51	HPV 59	100%	96.92%	MZ305439	HPV 51	81.2
6	G 42	HPV 45	99%	92.62%	MZ305444	HPV 42	81.3
		HPV 11	99%	93.29%	MZ305443		
7	5BA	HPV 6	99%	97.64%	MZ305446	HPV 59	81.4
8	G 83	HPV 59	98%	98.40%	MZ305440	HPV 83	80.6
9	4 BA	HPV 16	100%	97.92%	MZ305447	HPV 53	80.9
		HPV 16	100%	91.84%	MZ305448		
10	G 40	HPV 11	88%	98%	MZ305442	HPV 40	81.7
11	G 43	HPV 6	92%	98%	MZ305445	HPV 43	81.4
12	IR-M 12	HPV 16	91%	97%	MG825052	HPV 16	80.9
13	IR-EA13	HPV 16	99%	98.79%	MG825051	HPV 16	
26	IR-BA18	HPV 16	99.55	98.24%	MG825053	HPV 16	
14	IR_MSH18	HPV 18	98%	95%	MG825061	HPV 18	81.4
16	IR-EA10	HPV 66	99%	100%	MG825049	HPV 66	79.9
22	IR-M153	HPV 66	100%	95%	MG825050	HPV 66	
18	IR-M 15	HPV 89	89%	98%	MG825062	HPV 89	82.7
19	IR-K 69	HPV 52	95%	97%	MG825054	HPV 52	83
20	IR.BA.Mix1	HPV 58	98%	91%	MG825055	HPV 58	–
21	IR.BA.Mix2	HPV 58	97%	93.5%	MG825056	HPV 58	
23	IR-K38	HPV 6	100%	97.10%	MG825059	HPV 6	81.3
24	IR-M15-6	HPV 6	100%	100%	MG825058	HPV 6	
25	IR-K36	HPV 6	100%	100%	MG825057	HPV 6	
15	IR-MSH6	HPV 6	100%	100%	MG825060	HPV 6	
27	IR-K76	HPV 61	100%	99.74	MG825048	HPV 61	–

**Figure 1:**
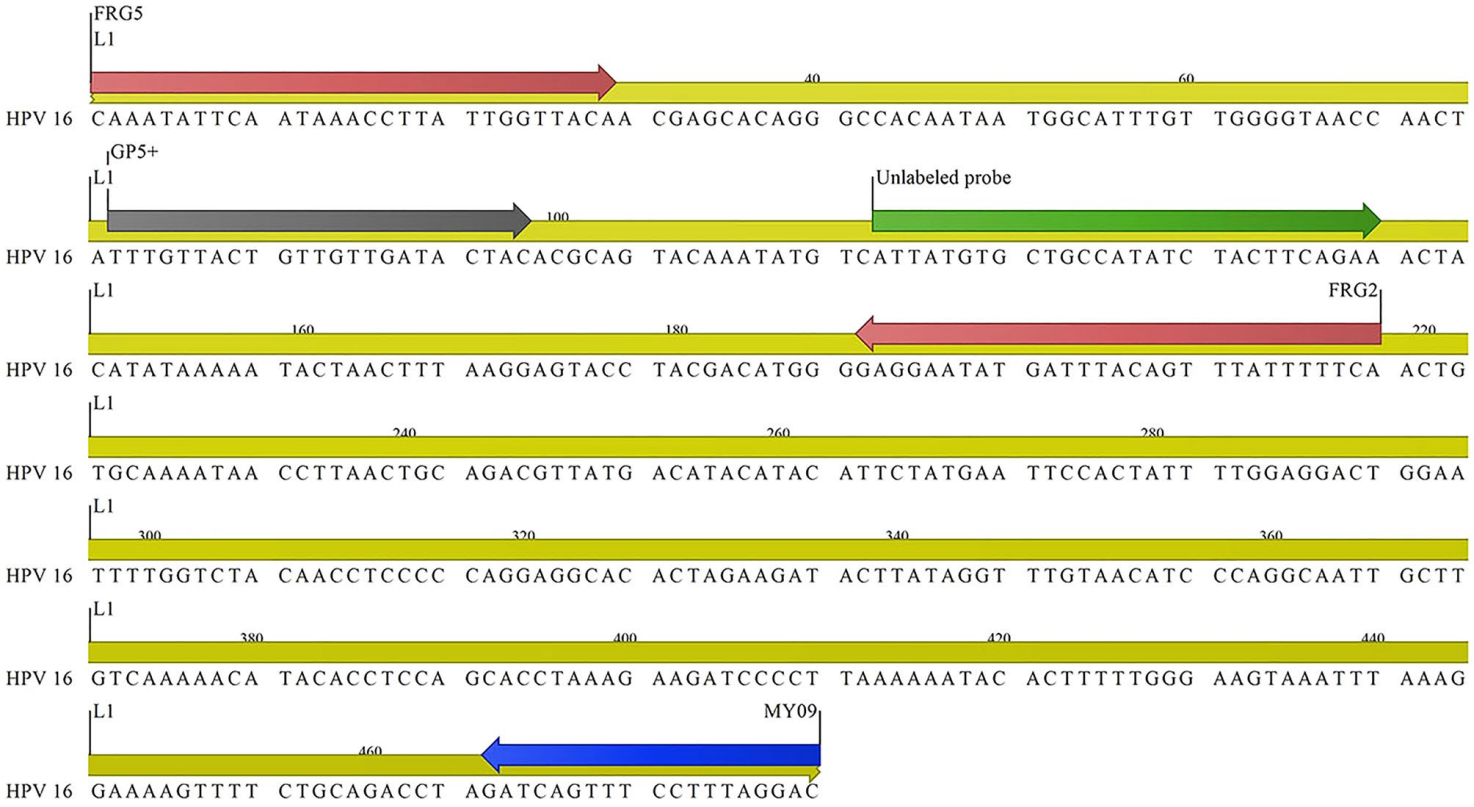
Schematic map of the *L*1 region human papillomavirus, primers and unlabeled probe using CLC workbench 12 software.

**Figure 2:**
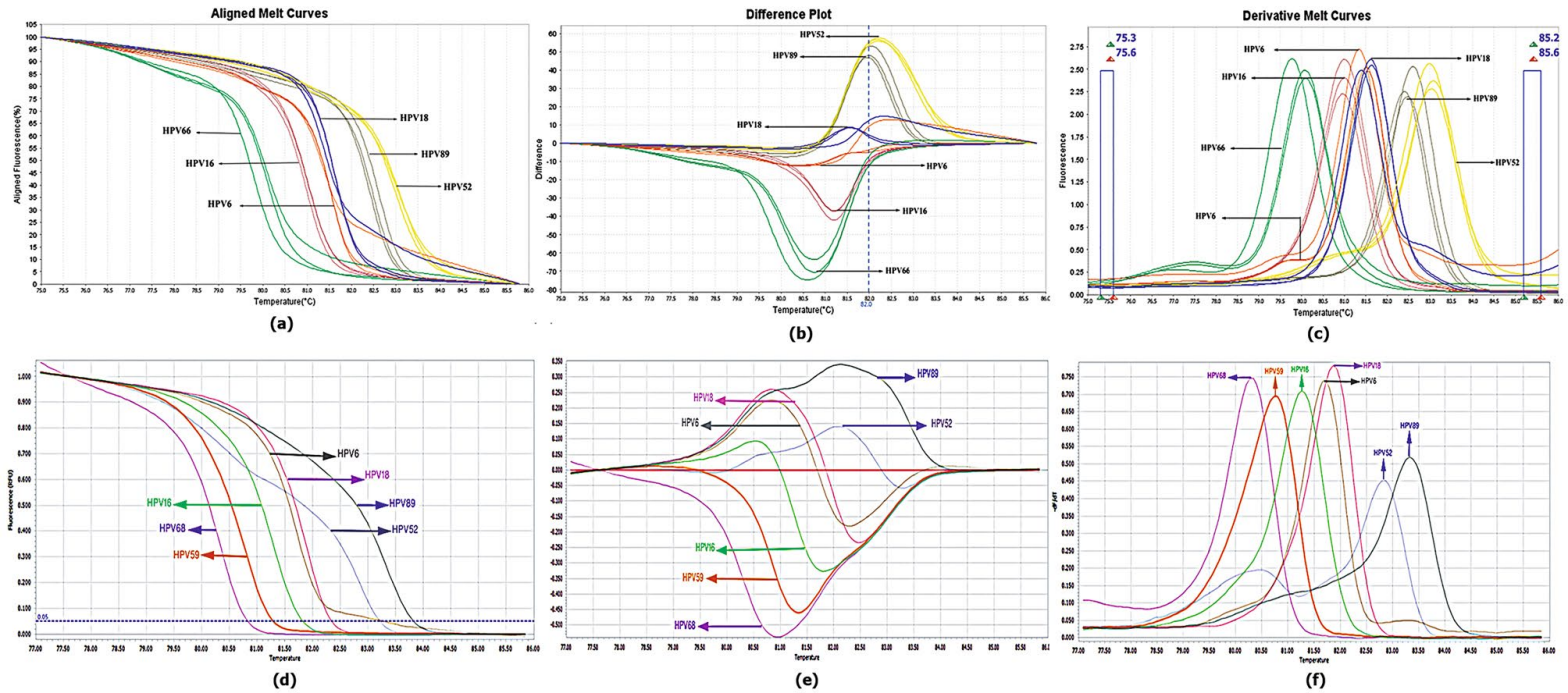
Real-time PCR of the *L*1 amplicon of HPV 6, 16, 18, 52, 59, 66, 89 using the primers FRG5 and FRG2. Aligned melt curves, difference plot, and derivative melt curve on StepOnePlus real-time PCR system receptivity (**a**,**b**,**c**). Aligned melt curves, difference plot, and derivative melt curve on a LightCycler 96 machine, receptivity (**d**,**e**,**f**).

Semi-nested qPCR-HRM by FRG5/2 primers showed that Tm could discriminate among HPV 16, 52, 59, 66 and 89. While HPV16, 52, 59, and 66 are classified as high risk, HPV89 is classified as low/no risk. HPV 18 and HPV 6 showed however very similar melting curve profiles as predicted in Table 1 (Figs. 1, 2), despite showing 61 base differences and 72.4% nucleotide similarity in the selected FRG2/5 region, showed similar melting curve profiles which was in accordance with the predictions in Table 1.

**Figure 3:**
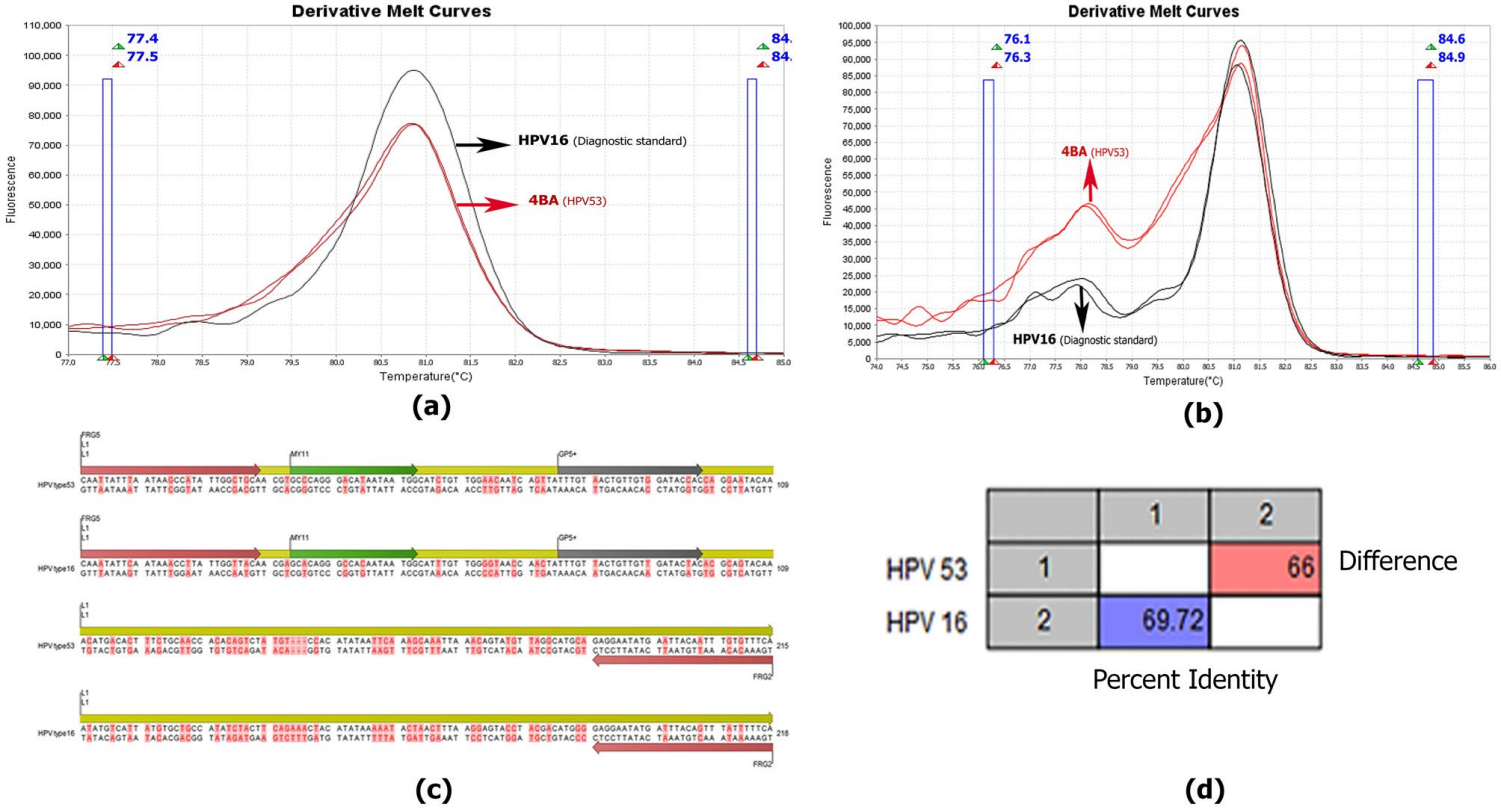
Derivative melt curve released of approach one, related to HPV 16 as a diagnostic standard and isolate 4BA as microarray-identification HPV 53 (**a**). Derivative melt curve released of approach two, related to HPV 16 as a diagnostic standard and isolate 4BA as microarray-identification HPV 53 (**b**). Schematic map of the amplification region of human papillomaviruses 16 and 53 in this study and the location of the primers using CLC workbench 12 software (**c**). Pairwise comparison of the amplified region in approach one related to HPV 16 (diagnostic standard) and isolate 4BA (HPV 53) using CLC workbench 12 software (**d**).

The findings in first approach using HPV-positive samples genotyped by the microarray method and diagnostic standards were inconsistent with our expectations. For instance, HPV DNA genotyped HPV53 using the microarray technique was compared to our diagnostic standards and innovative technique nested-high resolution melting (Fig. 3).

**Figure 4:**
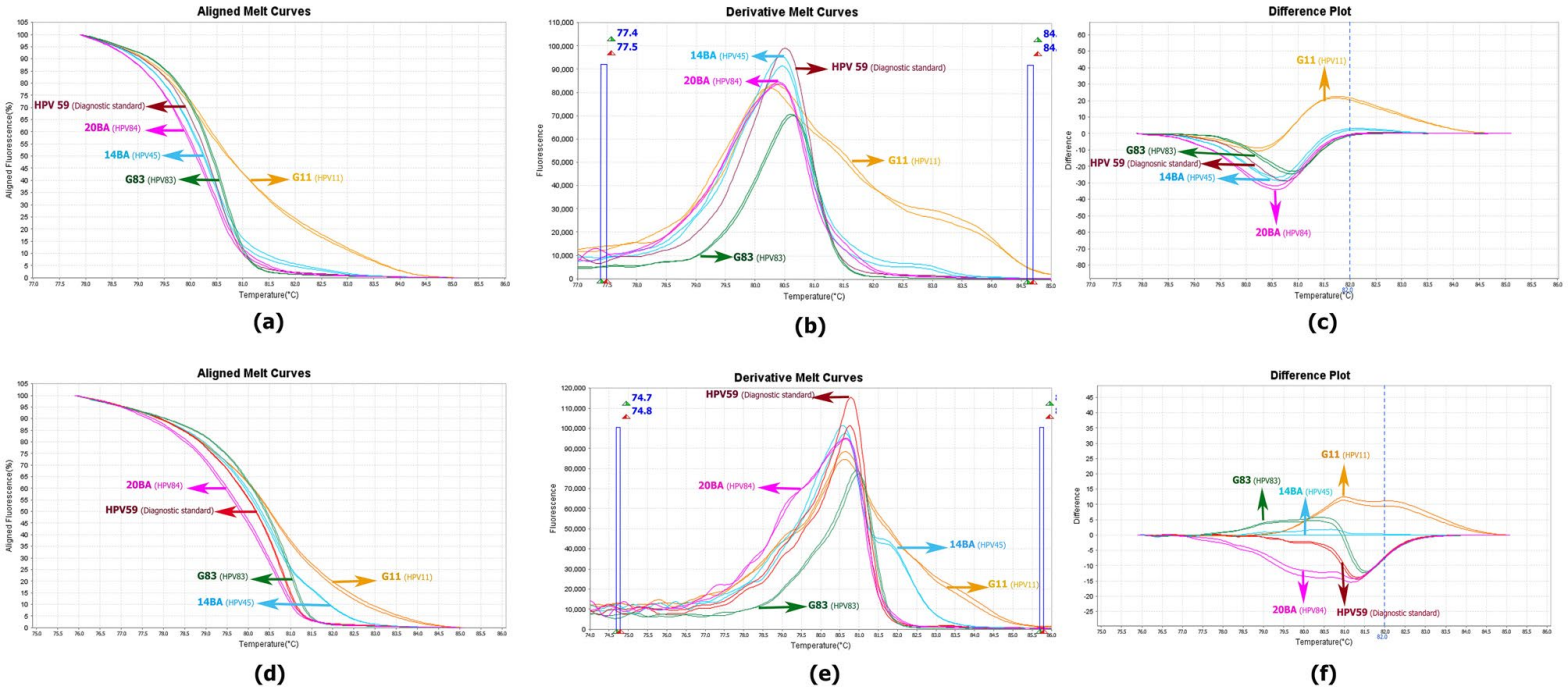
Aligned melt curves, derivative melt curve, and difference receptivity in approach one related to HPV 59 (diagnostic standard) and DNA HPV identified by the microarray method (**a**,**b**,**c**) Aligned melt curves, derivative melt curve, and difference receptivity in approach two related to HPV 59 (diagnostic standard) and DNA HPV identified by the microarray method (**d**,**e**,**f**).

As a result, the microarray-identification genotypes HPV 45, 59, 84, 11, and 83 all had melting curve profiles similar to HPV 59 diagnostic standard (Fig. 4).

**Figure 5:**
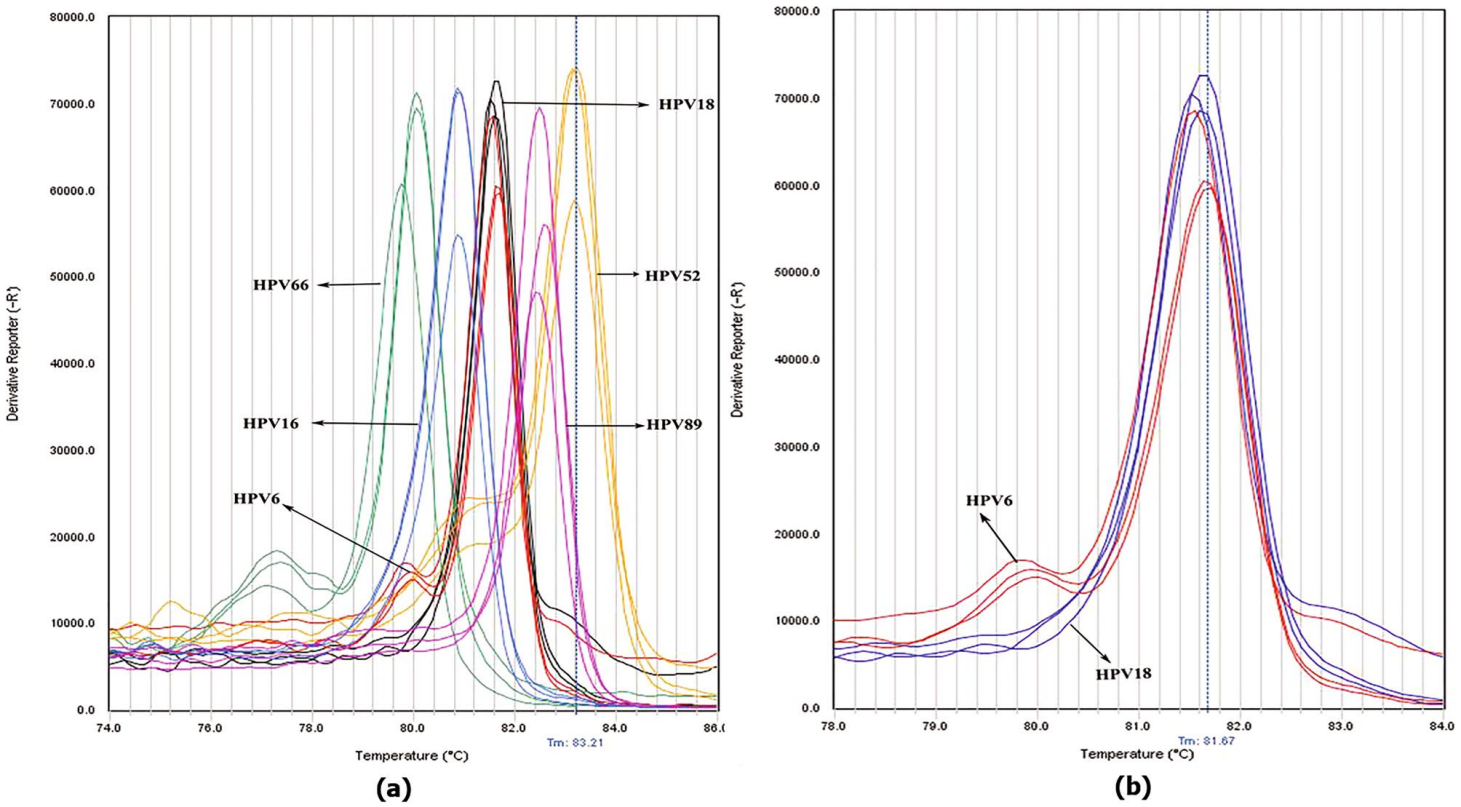
Melt curve of semi-nested qPCR-HRM reaction with FRG5, FRG2, and unlabeled probe U18 in approach three (**a**) related to diagnostic standard (**b**) HPV18 next to HPV6.

To distinguish between HPV 18 and 6, the unlabeled prob18 was added to the reaction mixture, and the test was repeated as the second approach. The observation of two peaks in the HPV6 plasmid enabled discrimination from HPV18 (Fig. 5).

**Figure 6:**
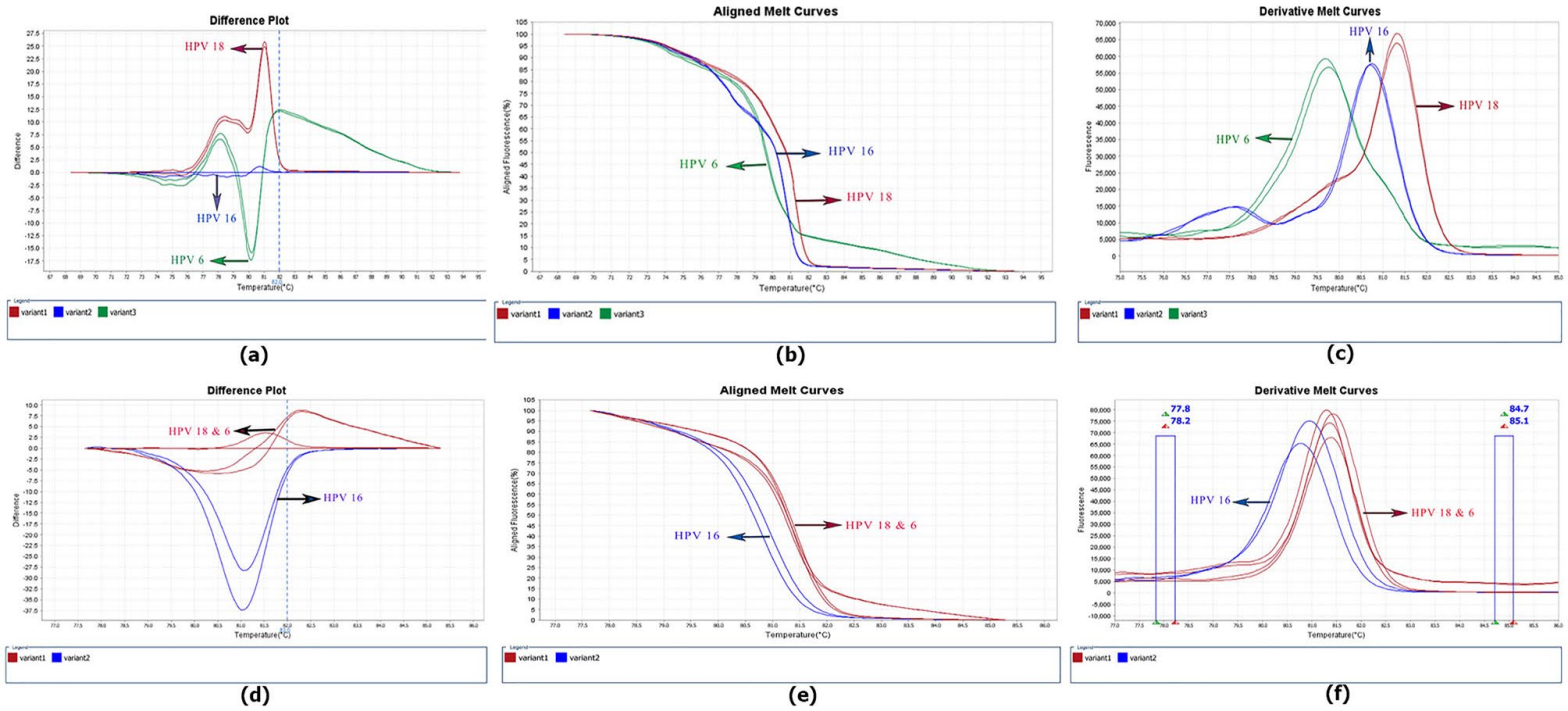
Difference plot, Aligned melt curves, and Derivative melt curve of semi-nested-qPCR-HRM HPV reaction for discrimination of human papillomaviruses with FRG5, FRG2, and GP5 primers in approach two (**a**,**b**,**c**). Difference plot, Aligned melt curves, and Derivative melt curve of semi-nested-qPCR-HRM reaction for discrimination of human papillomaviruses with FRG5, FRG2, and without GP5 in approach one (**d**,**e**,**f**).

In third approach, we added GP5 + to the reaction mixture to observe the effect of this single primer on the discrimination of HPV genotypes. Surprisingly, FRG2/FRG5 and GP5 + primers discriminated HPV 6 from HPV18 (Fig. 6). In addition, the third approach was able to differentiate the other 5 genotypes similar to the first and second approaches. This approach was used for the HPV positive samples identified by microarray, and observations showed that the results were reproducible.

Sample 4BA, identified as HPV53 based on microarray, had temperature and Tm characteristics similar to HPV 16 diagnostic standard by approaches one and three that used HRM technique. As shown in Table 3, the genotype of sample 4BA was confirmed to be HPV16 by Sanger sequencing (Fig. 4), and HPV 45, 59, 84, 11 and 83 had a similar pattern in method 1 (Fig. 3).

### Evaluation of sequencing for HPV genotyping

To clarify the contradictions which arose from the innovative technique nested-high resolution melting and microarray, PCR products from the first approach on HPV DNA were sent for sequencing. Sequences were blasted in NCBI (Table 3).

Sequence from Isolate 4 BA (which had been genotyped as HPV 53 using microarray and HPV 16 in the nested-qPCR-HRM) revealed the highest similarity to HPV 16 type when performing blast. Bioinformatics analyses of the 218 bp and 215 bp FRG5/2 amplicons from HPV16 and HPV53 showed a 30.28% nucleotide difference, which was significant enough to not be considered a sequencing or blasting error (Fig. 4).

Isolate G45 which was genotyped as HPV45 by microarray and had a similar temperature pattern to the diagnostic standard of HPV 59, was established as human papillomavirus 59 after blasting the sequencing results in the NCBI database (MZ305436). The two genotypes of human papillomavirus at the salt concentration of 0.2 in the FRG5/2 diagnostic region have the same melting temperature, differences in 61 bases, and 72.77 percent identity.

**Figure 7:**
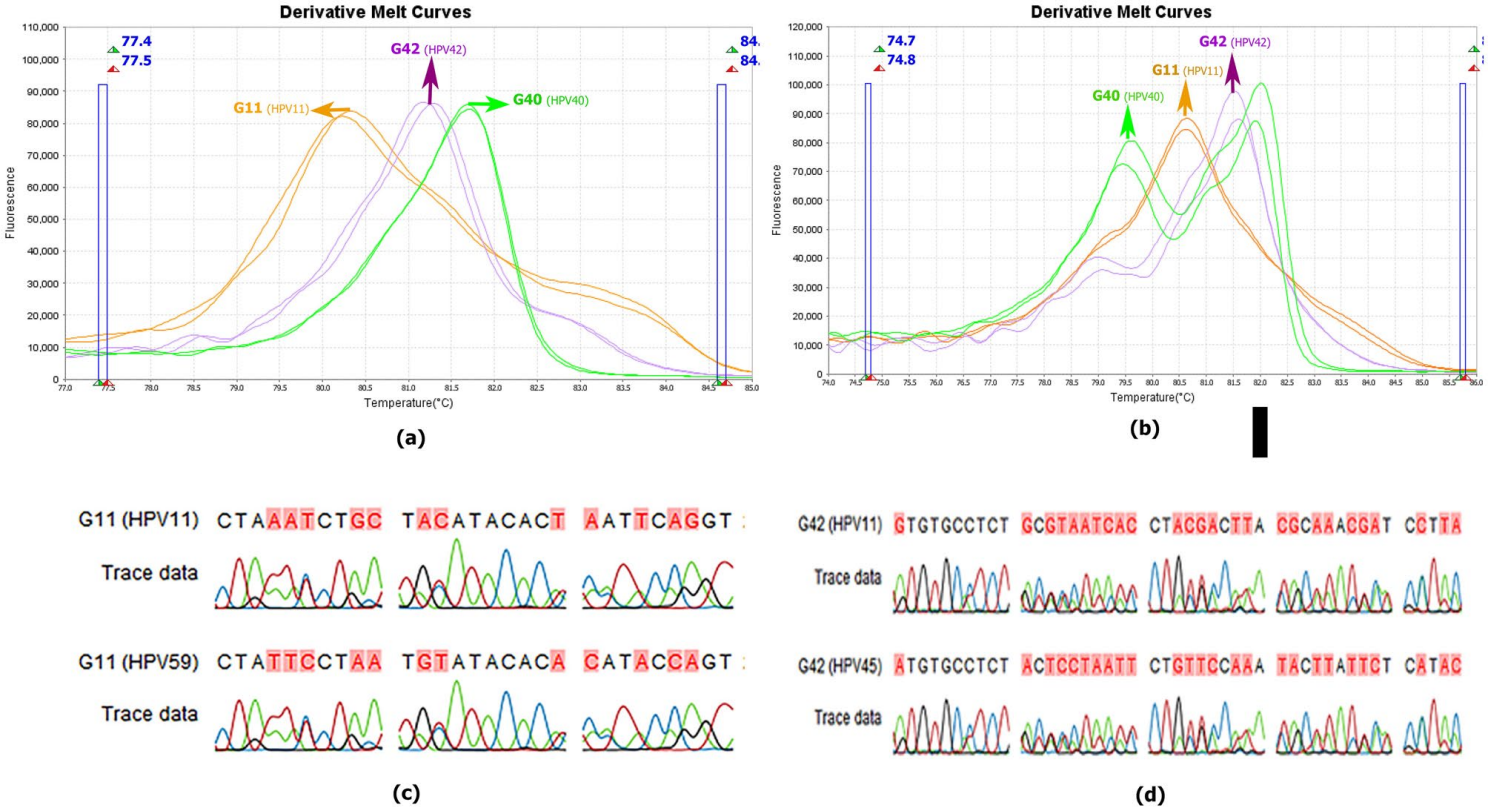
The derivative melt curve released of approach one is related to human papillomaviruses of isolates G11, G42, and G40 (**a**). The derivative melt curve released from approach two is related to isolates G11, G42, and G40 (**b**). Schematic map of the sequencing result of amplification region G11 and the presence of HPV mix infection after editing the sequence using CLC workbench 12 software (**c**). Schematic map of the sequencing result of amplification region G42 and existence of HPV mix infection after editing the sequence using CLC workbench 12 software (**d**).

As shown in Table 3, isolates G83 and 20BA, which were genotyped by microarray as HPV 83 and HPV 84, using Sanger sequencing were genotyped HPV 59, while bioinformatically the amplification region with the FRG2/FRG5 primers in HPV83 and HPV84 had 69.68% and 75.11% similarity to HPV 59, respectively. HPV DNA of isolate G11 after editing sequences and blasting in NCBI was considered a mixture of HPV 11 and 59, which had a similar melt curve in both approaches 1 and 2, despite the compliance of the graph peak and same Tm with the diagnostic standard HPV 59 showing a different pattern (Fig. 3). The two genotypes have a difference in 65 bases, and 70.72% similarity in the diagnostic area, and bioinformatics predicted that they be discriminated by a difference of 0.6 °C in melting temperature. HPV42 which was genotyped by the microarray method obtained from the G42 isolate was considered a combination of types 11 and 45 after sequencing and evaluation in the NCBI database (MZ305443-4). Genotypes 11 and 45 each have 72.09 and 71.88% identity to genotype 42, respectively. Besides, HPV DNA from isolate G 40, which was identified by the microarray HPV 40, was genotyped by sequencing HPV 11. The melting temperature of the two genotypes differed by approximately 1.5 degrees in terms of bioinformatics, and their sequence in the amplification area under study had 71 base differences and 68.30% identification (Fig. 7).

**Figure 8:**
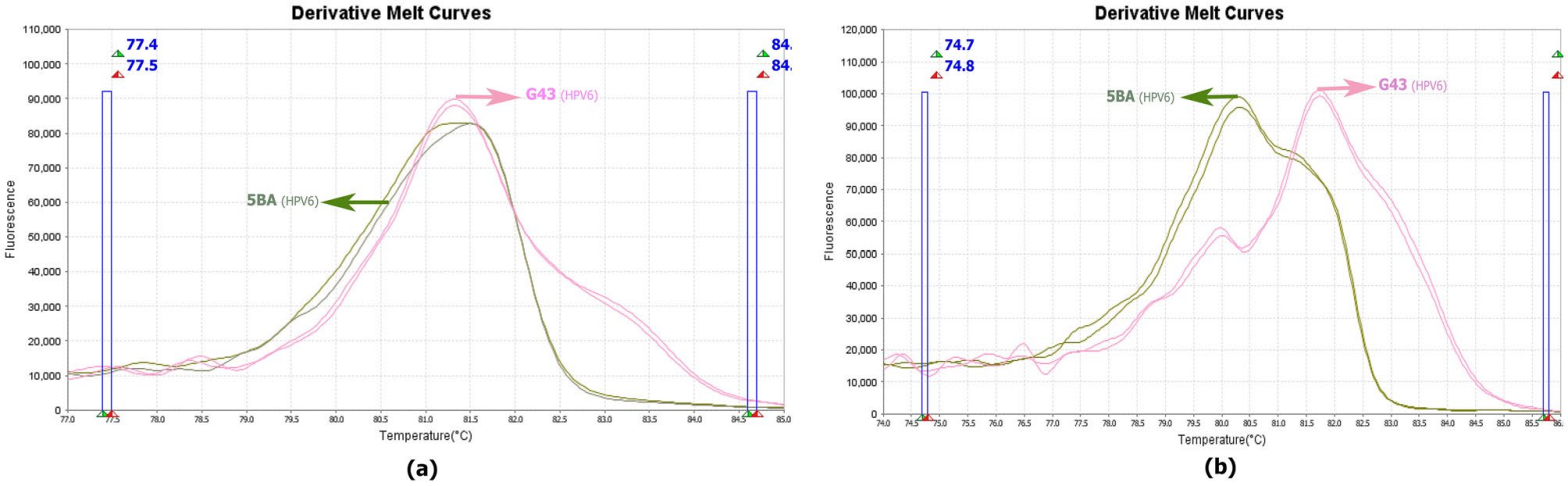
The derivative melt curve released from HPV discrimination using approach one is related to isolates 5BA and G43 (**a**). Derivative melt curve released from HPV discrimination using approach two, related to isolates 5BA and G43 (**b**).

5BA and G43 isolates, which were identified as HPV 59 and 43, respectively, were isolated by the microarray method. After sequencing, the reading result of both isolates was HPV 6. The first approach to examine these two samples showed the same melt curve, but in the second approach, the graphs were different (Fig. 8).

## Discussion

Due to the importance of identifying low-risk genotypes, high-risk genotypes, and mixed infections, a rapid and cost-effective method is needed to address HPV genotyping obstacles.

Cervical cancer is the second most common malignant tumor in women and seriously threatens women's health. Although cervical cancer is preventable, more than 500,000 women worldwide are diagnosed with cervical cancer, and more than 250,000 women die of cervical cancer each year^26^. According to World Health Organization (WHO), human papillomavirus is currently the most common sexually transmitted disease in terms of the unprotected sex, and the most important consequence of the virus is cervical cancer^27, 28^.

Diagnosing precancerous cells that are part of cervical cancer screening can be invaluable. Therefore, cervical cancer screening for eligible and high-risk individuals, as well as the discrimination against HPV genotypes, is crucial in the prevention, diagnosis and early treatment of the disease^29^. Therefore, in addition to the screening methods, discrimination among HPV-DNA genotypes and determining the number of copies could be important, which was the main topic of our study. It is known that the number of copies of HPV infection determines the duration and severity of the illness^30^.

Hence, considering the above mentioned factors, melting-based curves, especially HRM, can be used as an alternative method in the simultaneous identification and discrimination of HPV genotypes. Furthermore, the acquisition of a novel technique by targeted selection of primers gives acceptable results and may be useful for discriminating between different genotypes.

Previous studies showed that HRM method could diagnose different subtypes of influenza A^31^, astroviruses^32^, *C. meleagridis*^23^, and Yersinia pseudotuberculosis^33^. Besides, melting curve alteration in HRM method discriminated Iranian *Leishmania* parasites of *L. major*, *L. tropica* and mixed infection in the study of Ghafari SM et al.^34^. In addition, Mosawi SH et al., on asymptomatic malaria status in eastern Afghanistan, could distinguish *P. vivax*, *P. falciparum*, and mixed infections using high-resolution melting analysis^35^. The differentiation of *Mitragyna* speciosa from allied *Mitragyna* species was performed using DNA barcoding‑high‑resolution melting analysis by Chayapol Tungphatthong et al.^36^.

This study could identify important epidemiological and carcinogenic HPV genotypes using the seminested-HRM approach. These findings were very promising because they provided acceptable results with less time and cost than conventional methods in the market.

The separation of two main carcinogenic genotypes, HPV18 and HPV16, as well as the predominant genotype, HPV 6, in the research region, was a significant hurdle in our investigation^37^. Preliminary studies on temperature melting analysis of these three genotypes with insilico assays showed that bioinformatically, it is impossible to separate HPV18 and HPV6 despite the difference in six nucleotides among these two genotypes in studied gene region with a temperature difference of 0.03 °C. However, HPV16 could be distinguished with a Tm = 82 °C but a temperature difference of 0.7 °C from HPV6 and HPV18. Moreover, in the firs approach of the study, non-considering in-vitro results, there was consistency with the bioinformatics analysis. The melting temperatures of HPV18, HPV16, and HPV6 were 81.67 °C, 81.04 °C, and 81.52 °C, respectively. HPV18, a high risk type being the second most common type found in cervical cancer and, HPV6—a low-risk genotype being the most common genotype in our study population^37^, did not show a unique profile and therefore were not distinguishable from one another. However, five HPVs, 16, 52, 59, 66 and 89, with unique characteristics were distinguished. To address this issue, and in light of the findings by Lee et al., we employed an unlabeled probe for HPV18 in the second method with the goal of differentiating HPV18 from HPV6, although the results were surprising. In contrast to Lee et al. investigation, .'s in which an unlabeled HPV-18 probe resulted in an extra melting peak for HPV18 that separated it from HPV456^6^, unlabeled probes for HPV6 had additional melting points; therefore, HPV6 and HPV18 could indefinitely separate, where this contradiction is still unknown.

Although the results of our approaches with the sequencing method as the gold standard have shown our high success to design this assay, comparing the samples identified using the microarray method with our study method has provided new challenges. However, the discrepancy among the microarray results and these two methods is still debatable because DNA microarray method has sufficient accuracy to detect known HPV subtypes simultaneously.

This challenge occurred for the isolates which were reported as HPV 11, 45, 83 and 84 using the microarray method, whose melting temperature curves were similar to the HPV 59 diagnostic standard in the semi-nested-qPCR-HRM method in the first and third approaches which were genotyped by the sequencing method as HPV59. According to these views, incorrect genotyping or inaccurate differentiation between low-risk genotypes (HPV11 and HPV84) and high-risk genotypes (HPV45, HPV83, and HPV59) might have irreversible effects^38^.

Another notable point is that although all HPV-DNA samples from isolates G40, G11 and G42 were genotyped by sequencing as HPV11, they did not show identical melting curves. This may be because, in only G40, HPV11 was a single infection, but in G11 and G42, HPV-DNA was a mixed infection. The predominant genotypes of these isolates were identified as 59 and 45, respectively.

In the case of G11 isolates, the presence of HPV-DNA 11 and 59 was genotyped after sequencing. In approach one, the different HRM pattern of this isolate compared to other isolates that confirmed the presence of HPV-DNA 59, despite having the same peaks, could prove the hypothesis of mixed infection. This justifies the discrepancy between the microarray results with the nested-high-resolution melting and sequencing methods.

In the third approach, the patterns were almost identical to approach one, with the addition of GP primers, confirming the difference between G11/HPV-DNA isolation and HPV59 as a pure diagnostic standard. Comparing all three methods, nested qPCR-HRM, Sanger sequencing, and microarray, showed that nested qPCR-HRM approaches almost corresponded to Sanger sequencing as the gold standard diagnostic.

These contradictions were observed in other studies; for example, in the study of Alexander Harlé et al. one sample had HPV 6/11 DNA, which was detected with conventional PCR and not with the Cobas assay. Besides, one sample had HPV 16 DNA detected with Cobas assay and not with conventional PCR, one sample had HPV High-Risk DNA which was detected with conventional PCR and not with Cobas assay, one sample had HPV 16 DNA detected with Cobas assay and HPV 16 and HPV HR DNA with conventional PCR, one sample had HPV 16 DNA detected with Cobas assay and not with conventional PCR and one sample had HPV 18 DNA detected with Cobas assay and not with conventional PCR. The different reason could be the absence of consensus probes designed by the Cobas assay^39^. However, several HPV genotyping assays have recently been reported that are capable of typing a relatively large spectrum of HPV genotypes, but they cannot be automated or deployed in a high-throughput platform^13, 40–43^.

In population-based cervical screening, human papillomavirus (HPV) types 16, 18, 31, 33, 45, and 52 are associated with 85% of HPV-associated cervical cancers^44^, and we anticipated that we would be able to clone more genotypes as the diagnosis standard, the discrepancy among the results obtained by the microarray method and the sequencing prevented us from achieving this goal. Genotypes cloned in this study represent common low-risk and high-risk HPVs in the Middle East^37, 45^. In this regard, the study of Lee et al. showed eight HPV genotypes 16, 18, 39, 45, 52, 56, 58, and 68 with a prevalence of over 75% in Asia, Europe, and the United States. Considering the limited genotypes of HPV preserved in our study, large-scale typing was limited for different HPV genotypes. It is expected that by obtaining more genotypes in the future, this method could be further evaluated and analyzed.

In conclusion, we evaluated the validation of HPV genotyping via Tm value and HRM analysis of nested real-time PCR, which displayed the differential melting curves of different human papillomaviruses.

This approach has the potential to improve the discriminating of seven HPV genotypes, including HPV 16 and HPV 18, as cervical cancer carcinogens. The assay may be suited for routine analysis to detect HPV DNA in molecular laboratories as an alternative to the Pap smear test and enables effective treatment management, which is very practical for the success of implementation of women's health programs in low-resource regions.

To set up this technique, it is necessary to check all the diagnostic standards that are foreseen in the kit with the all of samples which are in the workflow and to determine the HPV genotype using HRM technique, a decision is made based on the comparison of diagnostic standards Tm with the sample Tm. It is both simple and quick to perform which was shown to have high sensitivity and specificity. Furthermore, when used to screen samples, it can significantly reduce the cost and time.

## Method and material

### Study population and specimen collection

**Figure 9:**
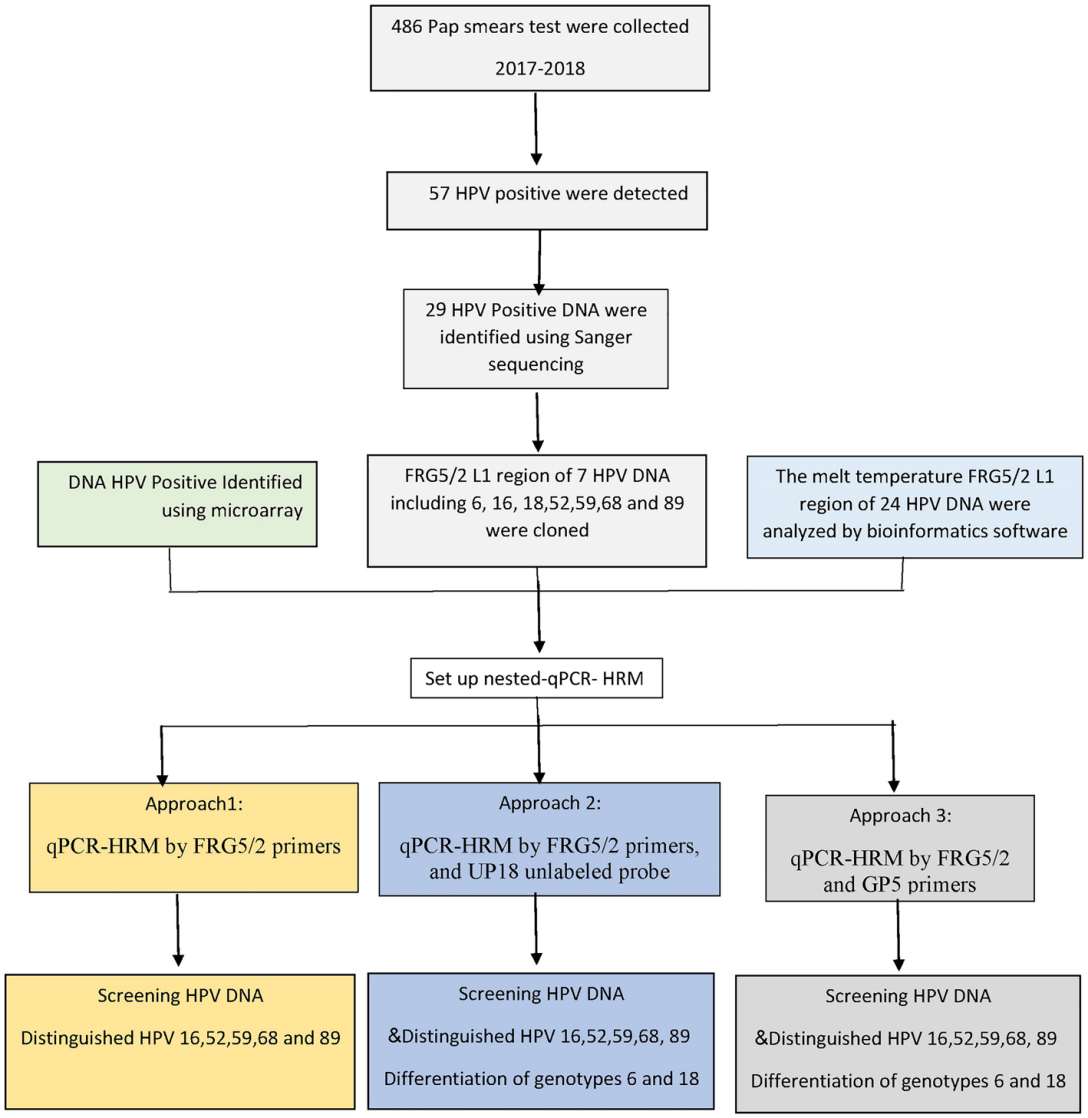
Flowchart of the study protocol.

The study protocol was reviewed and approved by Ethics Committee of Qom University of Medical Sciences (IR.MUQ.REC.1396.40), Iran, and all experiments were performed in accordance with relevant guidelines and regulations. A total of 57 HPV positive samples were randomly selected from 486 Pap smears analyzed in a previous study^37^. Informed consent was obtained from all women who were referred for conventional Pap tests to the medical centers of Qom Province. For optimization of the qPCR-HRM performance, known HPVs DNA were obtained from the previous study or microarray method in clinical laboratory^37^ (Fig. 9).

### DNA extraction and PCR

Cervical tissue DNA was extracted based on manufacturer’s protocol DynBio kit (Takapozist Co, Tehran, Iran). The quantity and quality of extracted DNA were measured by a Nanodrop One Spectrophotometer (Thermo Scientific, Wilmington, DE, USA) and the beta-actin gene, respectively (Table 2).

### Bioinformatics analysis and Study design

Establishing an effective qPCR-HRM approach requires choosing an area with a highly conserved region with the least interspecific variation for primer design and an unconserved region with the most intraspecific variation for pathogenic agent differentiation based on melting temperature.

For this purpose, the complete genomes of 24 HPV type-specific genomes were obtained from GenBank (Table 1). After multiple alignments of the sequences by CLC genomics workbench 12 (CLC, Bio-QIAGEN, Aarhus, Denmark), the most conserved region (*L*1) was preferred for assay design. The suggested primers were selected for ordering and establishment of the semi-nested qPCR-HRM method (Table 2).

Based on the selected primers, three approaches of qPCR-HRM technique were optimized for the identification of different HPV genotypes:

1. qPCR-HRM by FRG5/2 primers,
2. qPCR-HRM by FRG5/2 primers, and UP18 unlabeled probe,
3. qPCR-HRM by FRG5/2 and GP5 primers,

The melting temperature of different HPV genotypes was predicted using the “create sequence statistical analysis tool” in the CLC genomics workbench 12.

### Semi-nested-qPCR-HRM assay

For HPV detection, with the aim of screening and investigating HPV prevalence, 2 µl of the sample were subjected to PCR in a 20 µl reaction mixture volume using the general FRG5 forward and MY09 reverse primers on a conventional thermal cycler (Applied Biosystems, CA, USA) with the PCR program described in the reference 37. 29/57HPV-positive samples were sequenced (Bioneer, Korea) and submitted to GenBank (MG825048-MG825062 and MZ305435- MZ305448)^37^, and the other HPV-positive samples (28/57) were used for the three approaches mentioned below.

In the next step, to construct the diagnosis standard, identified HPV genotypes were amplified by conventional PCR using FRG5/2 primers. The 221 bp PCR product was cloned in the PTG19 vector according to the manufacturer's protocol of the Sinaclon kit (SinaClon, Tehran, Iran)^37^. All 7 plasmids were genotyped by Sanger sequencing (Bioneer, Korea) and then used in all approaches as diagnostic standards for HPV genotypes and amplification controls.

In the first approach, qPCR and HRM were performed in a single run by FRG5/2 primers in a reaction mix containing 4 μl 5 × Hot FIREPOL EvaGreen HRM Mix-Rox (Solis BioDyne, Estonia), 2 μl of diluted PCR product of FRG5/MY09 primers or plasmid, and 5 pmol of each primer with double distilled water to a total volume of 20 µl. The reaction conditions included an activation step at 95 °C for 15 min followed by 45 cycles of 95 °C for 20 s, 50 °C for 30 s, and 72 °C for 30 s. HRM was carried out over the range from 60 to 95 °C, with an increment of 0.3% °C for 15 s.

The second approach was performed with asymmetric qPCR and HRM by FRG5/FRG2 primers and UP18 probe. Briefly, the reaction mix contained 4 μl 5 × Hot FIREPOL Eva Green HRM Mix-Rox (Solis BioDyne, Estonia), 2 μl of diluted PCR product of FRG5/MY09 primers or plasmid, 2 μl of FRG2 (5 pmol/µl), 0.4 μl FRG5 (2/5 pmol/µl) and 2 μl probe UP18 (5 pmol/µl). The amplification protocol was predenaturation for 15 min at 95 °C followed by 65 cycles of denaturation for 15 s at 95 °C, annealing for 20 s at 46 °C, and extension for 30 s at 72 °C. HRM was continued from 60 to 95 °C, with an increment of 0.3% °C for 15 s^6^.

In the third approach, the qPCR-HRM technique was performed with FRG5, FRG2, and GP5 primers. The qPCR mix reaction and amplification protocol were performed according to the first approach.

In all approaches, deionized water was used as non-DNA blank control, and a plasmid containing the 225 bp ITS2 region of *Leishmania* (without an HPV DNA insert)^46^ was used as a negative control.

Each approach in the first phase was set up by identified diagnostic standards and then evaluated alongside microarray samples of patients.

To investigate intra-assay and interassay reproducibility, all reactions were performed in duplicated form on Applied Biosystems StepOnePlus (CA, USA) and LightCycler 96 (Roche Diagnostics, Penzberg, Germany) in two different labs. Moreover, assay performance was assessed by comparing CLART HPV4 (Genomica, Madrid, Spain) and the microarray method.

### Statistical analyses

All statistical analyses were performed with MedCalc statistical software (version 18.6; MedCalc Software, Ostend, Belgium) and SPSS software (version 25.0; IBM Corp, Armonk, NY).

### Ethics approval and consent to participate

The study protocol was reviewed and approved by Ethics Committee of Qom University of Medical Sciences (IR.MUQ.REC.1396.40), Iran.

## Data Availability

All data generated or analysed during this study are included in this published article.
